# Yangxin Decoction combined acupuncture on blood lipid metabolism in *Qi* Deficiency and *Blood* Stasis type of Chest *Bi*-Syndrome: A protocol of systematic review

**DOI:** 10.1097/MD.0000000000021744

**Published:** 2020-08-21

**Authors:** Xiao-hong Yu, Xi-wen Yu, Qi Zhang, Yu-ping Wang, Guo-qiang Yu

**Affiliations:** a Second Department of Cardiovascular, The First Affiliated Hospital of Heilongjiang University of Chinese Medicine, Harbin; b Department of Acupuncture and Moxibustion, Changchun Medical College, Changchun; c Department of Endocrinology, Heilongjiang Traditional Chinese Medicine Academy of Sciences; d Fifth Department of Internal Medicine, Hanan Branch of the Second Affiliated Hospital of Heilongjiang University of Traditional Chinese Medicine, Harbin, China.

**Keywords:** acupuncture, Chest *Bi*-Syndrome, *Qi* Deficiency and *Blood* Stasis type, Yangxin Decoction

## Abstract

**Background:**

In recent years, clinical studies about Yangxin Decoction combined acupuncture (YXDA) for the treatment of *Qi* Deficiency and *Blood* Stasis type of Chest *Bi*-Syndrome (CBS-QDBS) has been increased, but the results are different. The aim of this study is to investigate the effect of YXDA on blood lipid metabolism (BLMB) in patients with CBS-QDBS.

**Methods:**

We will collect any randomized controlled trials that assess the effect of YXDA on BLMB in CBS-QDBS from PUBMED, EMBASE, Cochrane Library, PsycINFO, CINAHL, Allied and Complementary Medicine Database, and China National Knowledge Infrastructure. All of these databases will be searched from their initial time to the present. All language limitation will be imposed. Literature selection, information collection, and risk of bias assessment will be performed independently by two authors, respectively. All data analysis will be undertaken using RevMan 5.3 Software.

**Results:**

This study will summarize the systematic nature of the literature search and its methods for assessing study quality and analyzing all relevant outcome data. Considering the inconsistent results, this study will improve the existing evidence on the effect of YXDA on BLMB in CBS-QDBS.

**Conclusion:**

The findings of this study will present the latest evidence of YXDA on BLMB in patients with CBS-QDBS.

Study registration: INPLASY202070047.

## 1 Introduction

Chest *Bi*-Syndrome (CBS) in traditional Chinese medicine (also known as chest pain) is a common complaint and symptom of myocardial infarction (MI) in the craniological and emergency departments,^[1–4]^ which is the leading cause of mortality and morbidity globally.^[5,6]^ There are over 8 million emergency visits for chest pain in the United States, and about 30% to 80% of them are due to acute coronary syndrome (ACS).^[7,8]^ It comprises of both ST-elevation MI and non-ST-evaluation ACS.^[9]^ Study reports that it may be associated with abnormalities in the metabolism of body fluids and tissue, such as blood lipid metabolism (BLMB).^[10,11]^ Previous studies have reported that Yangxin Decoction combined acupuncture (YXDA) can be used for the treatment of *Qi* Deficiency and *Blood* Stasis type of CBS (CBS-QDBS).^[12–23]^ However, no systematic study investigates this issue. Thus, this systematic study aims to explore the effect of YXDA on BLMB in patients with CBS-QDBS.

## 2 Methods and analysis

### 2.1 Study registration

This study was registered on INPLASY202070047, and it is designed based on the Preferred Reporting Items for Systematic Reviews and Meta-Analysis Protocol statement guidelines.^[24,25]^

### 2.2 Inclusion criteria for study selection

#### 2.2.1 Types of studies

Randomized controlled trials (RCTs) of YXDA on BLMB in patients with CBS-QDBS will be included, irrespective of blind, publication time and language. However, laboratory study, observational study, and non-RCTs will be excluded.

#### 2.2.2 Types of participants

Participants (18 years or older) with confirmed diagnosis as CBS-QDBS will be included, regardless ethnicity, gender, and country.

#### 2.2.3 Types of interventions

In the experimental group, all participants received any forms of YXDA. However, any single administration of acupuncture or Yangxin Decoction will be excluded.

In the control group, patients underwent any types of therapies, but not any forms of acupuncture or Yangxin Decoction or YXDA.

#### 2.2.4 Type of outcome measurements

Primary outcome is chest pain, as measured by electrocardiogram or any relevant examination test.

Secondary outcomes include cholesterol, triglycerides, phospholipids, urine routine test, alanine aminotransferase, aspartate aminotransferase, creatinine blood test, blood urea nitrogen test, and any adverse events.

### 2.3 Literature search

The following electronic databases will be searched from their initial time to the present: PUBMED, EMBASE, Cochrane Library, PsycINFO, CINAHL, Allied and Complementary Medicine Database, and China National Knowledge Infrastructure. We will not impose any restrictions of language and publication status. To perform a comprehensive and systematic search, an experienced librarian will be invited to develop search strategies for all electronic databases. A detailed search strategy for PUBMED is shown in Table 1. Identical search strategies will be modified and used to the other electronic databases.

**Table 1:**
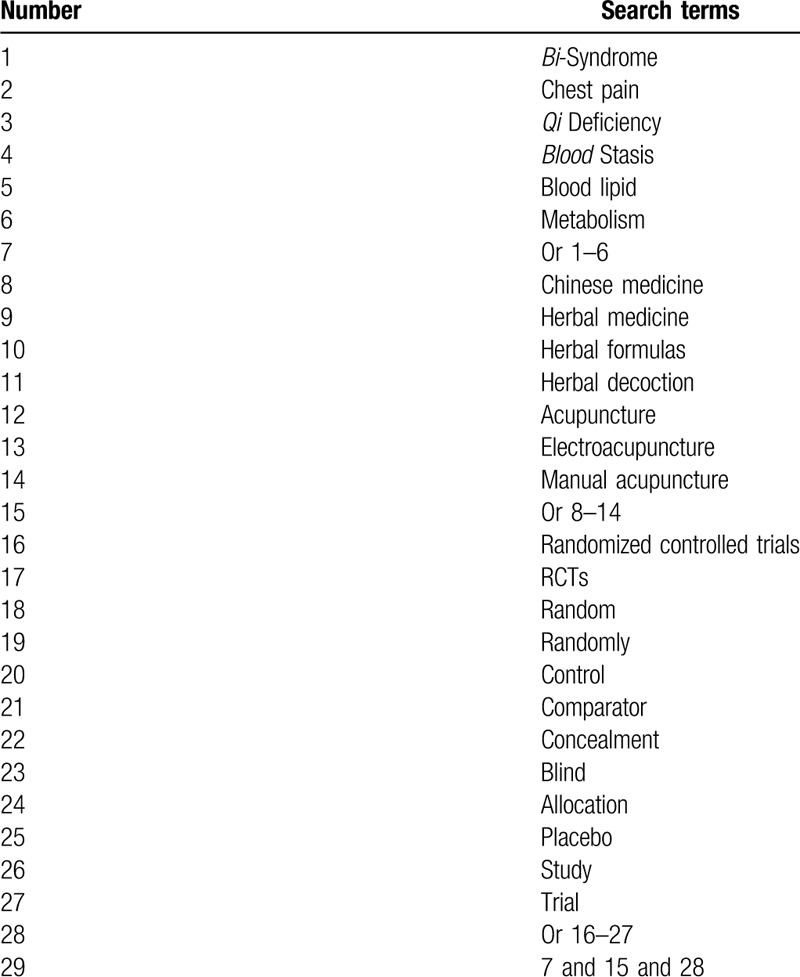
Search strategy for MEDLINE.

Moreover, we will identify conference abstracts, undergoing trials from clinical registry websites, and reference lists of relevant reviews.

### 2.4 Literature selection and data collection

#### 2.4.1 Literature selection

After all records were searched, the titles and abstracts of them will be reviewed by two independent authors to check potential trials based on the previously defined eligibility criteria. Then, we will read full-text of all potential trials to make sure whether they are eligible. We will record each excluded study with a specific reason. Any conflicts between two authors will be settled down by a third author through discussion. We will describe the selection of study process in a flow diagram.

#### 2.4.2 Data collection and management

Two independent authors will collect data based on the standardized form recommended by the Cochrane Handbook of Systematic Reviews of Interventions. Any conflicts between two authors will be solved with the help of a third author through discussion. The data collection form includes first author, title, year of publication, country, study setting, study duration, age, gender, diagnostic criteria, sample size, details of all experimental and control interventions, outcomes, funding and any other relevant data. Whenever necessary, if we identify any missing or unclear data, we will contact primary authors to request them.

### 2.5 Assessment of risk of bias in included studies

Two independent authors will assess the risk of bias for each qualified study using Cochrane Collaboration's “Risk of bias” tool in accordance with the guidelines of Cochrane Handbook for Systematic Reviews of Interventions. All risk of bias for each study will be checked through 7 aspects, and each one is graded as low, unclear or high risk of bias. Any conflicts between two authors will be figured out by a third author via discussion.

### 2.6 Statistical analysis

#### 2.6.1 Data analysis

Statistical analysis will be undertaken using RevMan 5.3 software. The dichotomous data will be expressed as risk ratio and 95% confidence intervals (CIs), while the continuous data will be presented as mean difference or standardized mean difference and 95% CIs. The level of heterogeneity among studies will be checked using *I*^2^ statistic. Reasonable heterogeneity will be regarded if *I*^2^ ≤ 50%, and we will employ a fixed-effects model, as well as meta-analysis performance if possible. Significant heterogeneity will be considered if *I*^2^ > 50%, and we will utilize a random-effects model. At the same time, we will operate subgroup analysis or meta-regression to explore possible causes for the substantial heterogeneity. In addition, summary results will be interpreted by providing detailed written commentary on the collected data based on the factors outlined in the data collection process section. It will advance our understandings of the YXDA on BLMB in patients with CBS-QDBS.

#### 2.6.2 Subgroup analysis

If there are adequate studies, we will handle the subgroup analysis based on the differences in interventions, controls, and outcomes.

#### 2.6.3 Sensitivity analysis

We will conduct a sensitivity analysis to check robustness of outcome results by excluding studies with high risk of bias.

#### 2.6.4 Assessment of reporting biases

We will run funnel plots and Eggers’ Regression test^[26,27]^ to check reporting bias if more than 10 qualified studies are included.

### 2.7 Ethics and dissemination

This study is a secondary analysis of published data; thus, no ethical approval is required. We plan to publish this study at a peer-reviewed journal or a conference proceeding.

## 3 Discussion

Traditional Chinese medicine, such as YXDA and acupuncture has been widely used to treat CBS-QDBS. However, its results are still inconsistent, and no study has been systematically carried out to check the effect of YXDA on BLMB in patients with CBS-QDBS. Thus, this study will specifically focus on investigating the effect of YXDA on BLMB in patients with CBS-QDBS. It may provide a detailed summary of the present evidence of YXDA on BLMB in CBS-QDBS. The results of this study may provide helpful evidence for both clinician and future researchers for the treatment of CBS-QDBS.

## Author contributions

**Conceptualization:** Xiao-hong Yu, Xi-wen Yu.

**Data curation:** Xiao-hong Yu, Xi-wen Yu.

**Formal analysis:** Xiao-hong Yu, Qi Zhang, Yu-ping Wang.

**Investigation:** Qi Zhang, Yu-ping Wang.

**Methodology:** Xiao-hong Yu, Xi-wen Yu, Qi Zhang.

**Project administration:** Qi Zhang, Yu-ping Wang.

**Resources:** Xiao-hong Yu, Qi Zhang, Yu-ping Wang, Guo-qiang Yu.

**Software:** Xiao-hong Yu, Xi-wen Yu, Qi Zhang, Yu-ping Wang, Guo-qiang Yu.

**Supervision:** Qi Zhang, Yu-ping Wang.

**Validation:** Xiao-hong Yu, Xi-wen Yu, Qi Zhang, Yu-ping Wang.

**Visualization:** Xiao-hong Yu, Xi-wen Yu, Guo-qiang Yu.

**Writing – original draft:** Xiao-hong Yu, Xi-wen Yu, Qi Zhang, Yu-ping Wang.

**Writing – review & editing:** Xiao-hong Yu, Xi-wen Yu, Qi Zhang, Guo-qiang Yu.
